# HLA molecular mismatch – A step towards precision medicine in transplantation for individualized immune risk assessment and immunosuppression

**DOI:** 10.3389/fmed.2026.1990225

**Published:** 2026-10-23

**Authors:** Christine Jia Yu Wong, Emmett Tsz Yeung Wong

**Affiliations:** 1 Department of Medicine, National University Hospital, Singapore, Singapore; 2 Department of Medicine, Yong Loo Lin School of Medicine, National University of Singapore, Singapore, Singapore; 3 National University Centre for Organ Transplantation, National University Hospital, Singapore, Singapore

**Keywords:** alloimmune risk, *de novo* donor-specific antibodies (DSA), eplet, immunosuppression, molecular mismatch

## Abstract

Despite advances in immunosuppression and histocompatibility assessment, late kidney allograft attrition remains an important challenge, with alloimmune injury a major cause of death-censored graft failure. More precise assessment of alloimmune risk could enable individualized immunosuppression and surveillance based on recipient risk. In this narrative review, we examine the evolution of histocompatibility assessment and the emergence of HLA molecular mismatch as a prognostic biomarker of alloimmunity. We review evidence linking molecular mismatch to *de novo* donor-specific antibody development, rejection and graft outcomes, and its interaction with immunosuppressive exposure. We critically evaluate potential applications to immunosuppression minimization, risk-adapted surveillance, and donor selection, while distinguishing prognostic validity from evidence for mismatch-guided intervention. Finally, we discuss methodological and biological limitations and emerging approaches integrating complementary molecular compatibility measures with functional and longitudinal biomarkers. Such multimodal strategies may ultimately enable more individualized assessment and management of alloimmune risk following kidney transplantation.

## 1 Introduction

Kidney transplantation is widely regarded as the optimal treatment for kidney failure and has been associated with improved patient survival, reduced cardiovascular mortality and events, and superior quality of life (1, 2). Over the last several decades, kidney transplantation outcomes have improved substantially, with significant gains in both short- and medium-term graft and patient survival. These improvements are largely attributable to advances in immunosuppressive therapy, donor-recipient matching, risk stratification strategies, improved organ preservation, and enhanced perioperative care (3). However, achieving durable long-term graft survival remains a significant challenge.

Long-term kidney transplant outcomes are determined by both graft and recipient survival. Matas et al. evaluated 2,202 kidney transplant recipients with at least 10 years of graft function and demonstrated that long-term outcomes remained suboptimal despite successful early transplantation (4). Among 10-year survivors, the major causes of late graft loss were death with a functioning graft and chronic allograft injury. Mayrdorfer et al., in a study of 1,477 adult kidney transplants, found that death with a functioning graft (DWFG) was predominantly driven by cardiovascular disease, malignancy, and infection, whereas death-censored graft failure (DCGF) was more strongly associated with alloimmune injury, particularly T-cell and antibody-mediated rejection (AMR). These findings highlight that graft loss reflects competing risks: under-immunosuppression leading to rejection and immunosuppression-related complications leading to infection and malignancy (5). Consequently, while early transplant success has improved considerably, further advances are crucial to address the mechanisms underlying chronic allograft dysfunction and extend long-term graft survival.

Human leukocyte antigen (HLA) compatibility has long been a critical determinant of successful transplant outcomes, with multiple analyses showing an association between HLA matching and improved long-term graft survival (6, 7). Historically, late graft failure was often attributed to chronic allograft nephropathy or calcineurin inhibitor toxicity.

More recently, Merzkani et al. retrospectively analyzed 5,752 kidney transplant recipients and found that graft failure in younger non-diabetic patients was predominantly attributable to alloimmunity, glomerular disease, and tubular injury, with alloimmunity accounting for approximately half of graft failures occurring beyond the first year (8). This is further supported by Mulley et al., who found that rejection was the leading cause of death-censored kidney allograft failure, accounting for 47.5% of 642 cases, with chronic antibody-mediated rejection responsible for 37.4% (9).

As AMR and transplant glomerulopathy emerge as major contributors to late graft failure, there is increasing recognition that more accurate assessment of immunological risk is needed to improve donor selection, personalize immunosuppression, and ultimately enhance long-term graft survival.

Recent advances in molecular immunogenetics have introduced the concept of molecular mismatch as a more precise measure of immunogenic disparity between donor and recipient (10, 11). Accumulating evidence demonstrates that higher molecular mismatch is strongly associated with the formation of *de novo* donor-specific antibodies (dnDSA) and AMR, and in several cohorts, provides greater discrimination of alloimmune risk than conventional antigen-level HLA mismatch.

Recent reviews have comprehensively described advances in molecular HLA compatibility assessment and the contribution of non-HLA genetic disparity to transplant outcomes (12). However, an important translational question remains: how can these increasingly precise measures of donor–recipient compatibility be incorporated into clinical risk assessment and, ultimately, individualized management? In this review, we therefore focus particularly on the clinical translation of molecular mismatch from a prognostic biomarker of alloimmune risk towards precision management in kidney transplantation. We examine its interaction with immunosuppressive exposure, its potential applications to immunosuppression minimization and post-transplant surveillance, and its integration with complementary molecular and longitudinal biomarkers. More precise risk stratification may ultimately help identify recipients who may require more intensive immunosuppression or closer surveillance, while supporting minimization strategies in appropriately selected low-risk individuals. We further consider recent developments in multimodal compatibility assessment and the evidence required before molecular mismatch-guided interventions can be incorporated into routine clinical practice.

### 1.1 Literature selection

This article is a narrative review and did not use a prospectively defined systematic search or a formal study-screening process. Relevant literature was identified primarily through searches of PubMed/MEDLINE using combinations of terms including ‘HLA molecular mismatch’, ‘eplet mismatch’, ‘HLAMatchmaker’, ‘HLA-EMMA’, ‘PIRCHE’, ‘kidney transplantation’, ‘donor-specific antibody’, ‘immunosuppression’, and ‘graft outcomes’, supplemented by reference-list screening and targeted searches for recent studies. Searches included literature published up to August 2026. We selected literature based on relevance to the review's major themes, with emphasis on seminal studies, larger contemporary cohorts, studies reporting clinically relevant alloimmune and graft outcomes, and studies evaluating potential clinical applications of molecular mismatch. We also considered recent literature on complementary molecular compatibility approaches and emerging clinical applications. Accordingly, this literature review is not an exhaustive systematic synthesis, and we acknowledge the potential for selection bias inherent to narrative review methodology.

## 2 Histocompatibility assessment in kidney transplantation

The successful kidney transplant between identical twins, performed by Joseph Murray in 1954, demonstrated the potential for sustained graft survival in the absence of donor-recipient genetic disparity (13). Subsequent advances in transplant immunology established the importance of histocompatibility antigens, leading to the development of HLA typing and donor-recipient matching (Figure 1).

**Figure 1:**
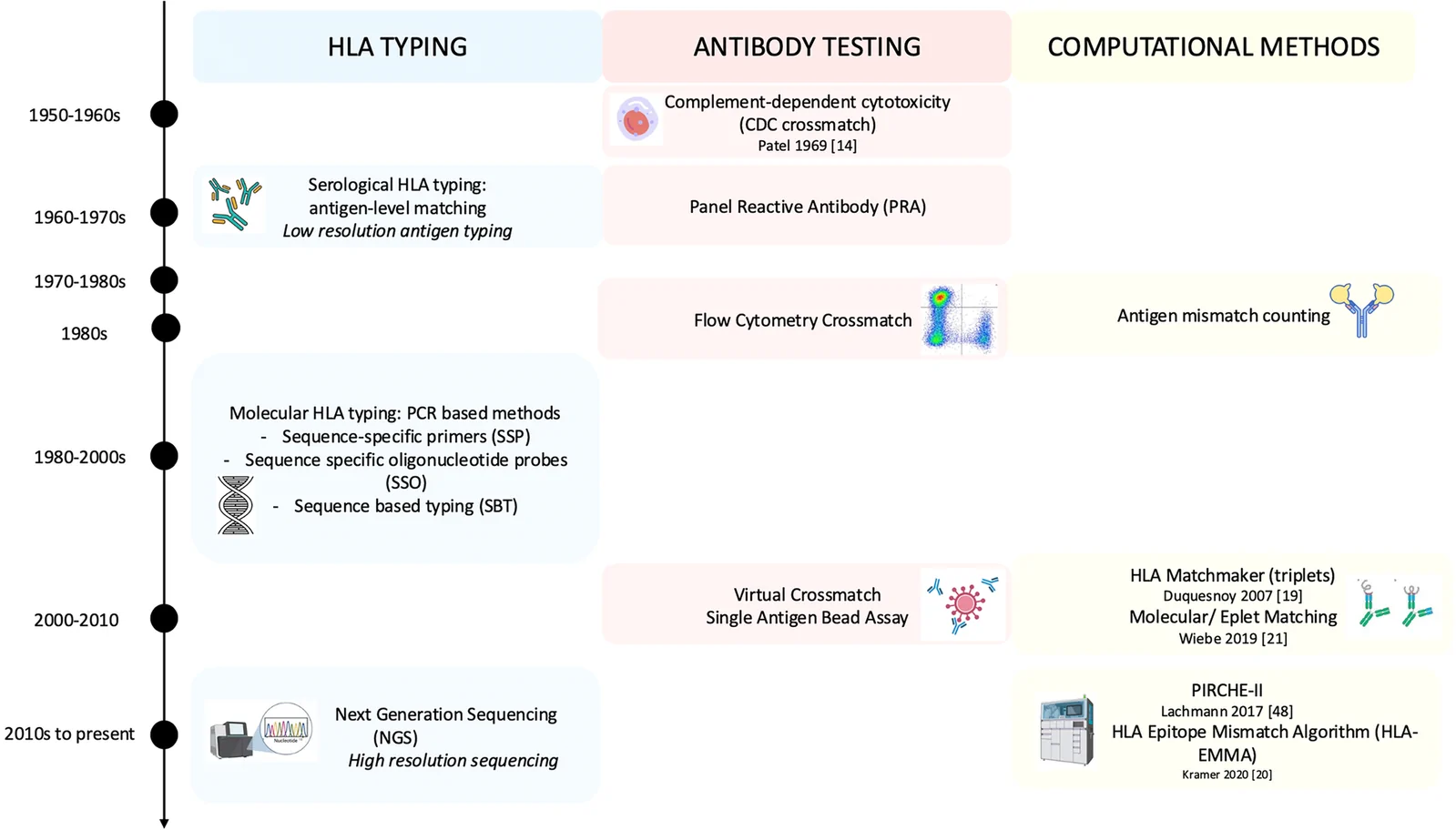
Timeline of histocompatibility assessment in kidney transplantation. Schematic timeline illustrating major developments in donor–recipient histocompatibility assessment across three complementary domains: HLA typing, antibody testing, and computational compatibility assessment. HLA typing evolved from serological antigen-level typing to molecular PCR-based methods and high-resolution next-generation sequencing. Antibody assessment progressed from complement-dependent cytotoxicity crossmatching and panel-reactive antibody testing to flow cytometry crossmatching, single-antigen bead assays, and virtual crossmatching. Computational assessment evolved from conventional antigen mismatch counting towards structurally and molecularly defined compatibility approaches, including HLAMatchmaker, molecular/eplet matching, the HLA Epitope Mismatch Algorithm (HLA-EMMA), and Predicted Indirectly Recognizable HLA Epitopes (PIRCHE-II). Selected landmark studies illustrating key developments are shown; these are representative rather than exhaustive. Timings indicate approximate periods of development and clinical adoption, and several methods remain in concurrent use.

While HLA typing and antigen-level matching provided an important means of assessing donor-recipient histocompatibility, they did not account for pre-existing recipient alloantibodies. In a landmark study of 225 kidney transplants, Patel et al. demonstrated that a positive complement-dependent cytotoxicity (CDC) crossmatch—detecting preformed cytotoxic antibodies against donor lymphocytes—was strongly associated with immediate graft failure, establishing prospective crossmatching as a critical component of pre-transplant immunological risk assessment (14, 15). As a population extension of the CDC approach, the panel reactive antibody (PRA) was subsequently developed—instead of testing against a single donor, the recipient serum was tested against a panel of lymphocytes from multiple donors. A high PRA percentage would suggest that a recipient was highly sensitized and thus had a higher likelihood of a CDC-positive crossmatch, implying a reduced likelihood of a donor offer at an acceptable immunological risk.

During the 1980s and 1990s, flow cytometry crossmatch emerged as a more sensitive cell-based method for detecting recipient antibodies binding to donor lymphocytes, including lower-level donor-reactive antibodies that may be undetectable by the conventional CDC crossmatch. When attributable to anti-HLA antibodies, such reactivity may indicate the presence of DSAs. The subsequent development of solid-phase assays represented another major advance in histocompatibility testing, enabling increasingly sensitive and specific characterization of anti-HLA antibodies. In particular, Luminex-based single-antigen bead assays (SAB) use beads coated with individual HLA antigens to identify antibody specificities that conventional cell-based crossmatching may not detect. These advances facilitated the development of the virtual crossmatch, whereby a recipient's anti-HLA antibody profile is compared with the prospective donor's HLA typing to predict immunological compatibility without requiring a physical crossmatch between recipient serum and donor lymphocytes.

### 2.1 Evolution from antigen-level to molecular histocompatibility assessment

While conventional HLA matching and antibody-based assessment remain central to immunological risk stratification, neither fully captures the alloimmune potential of an individual donor-recipient combination. SAB assays enable sensitive detection and characterization of circulating anti-HLA antibodies, although not all detected antibodies are clinically relevant, and the absence of detectable DSA does not necessarily exclude pre-existing humoral immune memory in previously sensitized individuals (16). Donor-reactive memory B cells may persist despite the absence of circulating DSAs and can rapidly generate an anamnestic response following re-exposure to matching allogeneic HLA (17).

Importantly, alloimmune risk is not confined to recipients with pre-existing antibodies or immunological memory. Donor–recipient HLA disparity itself provides the antigenic stimulus for primary alloimmunization, potentially leading to the generation of dnDSA and subsequent antibody-mediated injury. Conventional antigen-level HLA matching provides only a relatively coarse measure of this disparity, as HLA mismatches considered equivalent at the antigen level may differ substantially in their molecular composition and immunogenic potential (18). This limitation provided the rationale for increasingly granular approaches to histocompatibility assessment based on differences in the amino acid sequences and structures of donor and recipient HLA molecules.

An important step toward molecular approaches for histocompatibility assessment was the development of HLAMatchmaker by Duquesnoy. The approach was based on the principle that alloantibodies recognize discrete structural configurations of polymorphic residues on the surface of HLA molecules rather than HLA antigens as a whole. The original model characterized donor-recipient differences using polymorphic amino acid triplets exposed on the molecular surface of donor and recipient HLA molecules. This concept subsequently evolved from triplets to eplets—patches of surface-exposed non-self amino acid residues within a 3 Å radius which can form antibody paratope-HLA epitope interactions (Figure 2) (19). HLAMatchmaker identifies eplets present on donor HLA molecules but absent from the recipient's own HLA repertoire, thereby providing a quantitative measure of donor-recipient molecular disparity. Importantly, an eplet represents a computationally defined structural unit and should not necessarily be considered synonymous with an experimentally verified antibody epitope (Table 1).

**Figure 2:**
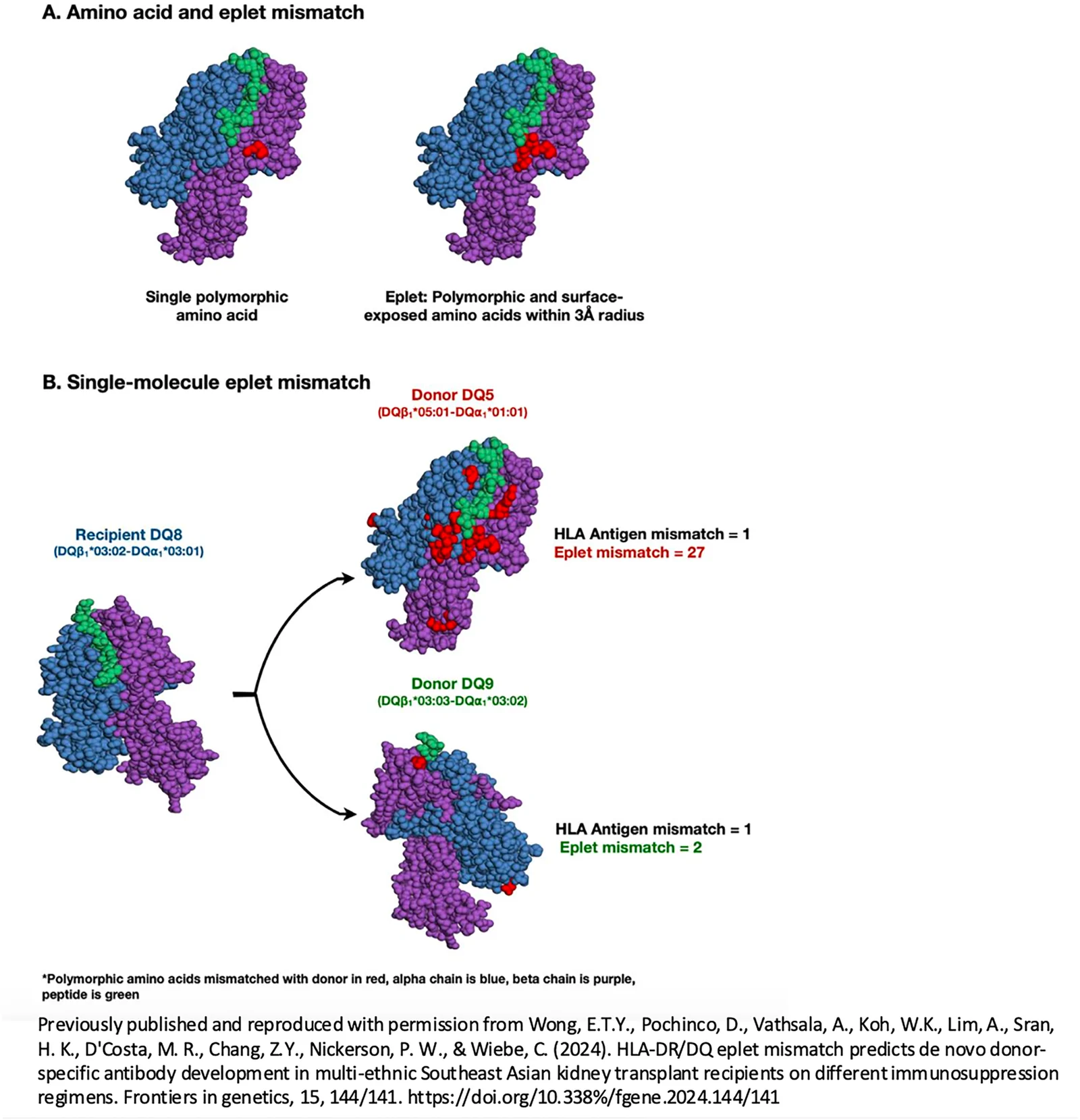
Amino acid, eplet, and single-molecule eplet mismatch. A mismatched polymorphic amino acid (red) is the most basic unit of HLA mismatch. Eplets are single or small patches of mismatched polymorphic amino acids within a 3 Å radius exposed near an HLA molecule's surface. Eplet mismatch has been recognized as a more precise strategy for determining HLA compatibility by enhancing the quantification of donor-recipient HLA difference at the molecular level (2A). A theoretical example of single-molecule eplet mismatch at HLA-DQ locus. The eplet mismatch for each donor HLA-DQα_1_*β*_1_ molecule is determined individually by comparing each molecule with all recipient HLA-DQα_1_*β*_1_ molecules. Two different HLA mismatches can have significantly different single-molecule eplet mismatches despite both having one HLA antigen mismatch. (2B) (Previously published and reproduced with permission from Wong, E. T. Y., Pochinco, D., Vathsala, A., Koh, W. K., Lim, A., Sran, H. K., D'Costa, M. R., Chang, Z. Y., Nickerson, P. W., & Wiebe, C. (2024). HLA-DR/DQ eplet mismatch predicts *de novo* donor-specific antibody development in multi-ethnic Southeast Asian kidney transplant recipients on different immunosuppression regimens. Frontiers in genetics, 15, 1447141. https://doi.org/10.3389/fgene.2024.1447141).

**Table 1:** Domains of histocompatibility assessment in kidney transplantation.

Domain	What is assessed	Tools/ tests	Interpretation	Clinical significance
ABO compatibility	Donor–recipient ABO compatibility and preformed anti-A/B antibodies	ABO blood group determination ± anti-A/B isohemagglutinin titres	Preformed anti-A/B antibodies can mediate hyperacute or acute AMR in the absence of appropriate desensitization	ABO-incompatible transplantation can be performed with desensitization but with potentially higher risks of rejection and complications including infections
HLA typing	Donor/recipient HLA type and mismatch	Class I: HLA-A, -B and –C Class II: HLA-DRB1/3/4/5, -DQB1, -DQA1, -DPB1, -DPA1	Increasing HLA mismatch is associated with greater alloimmune risk, although the immunogenicity of individual mismatches varies	Greater HLA compatibility is generally associated with lower risks of alloimmunization, rejection and graft loss.
Sensitization risk	Breadth of recipient sensitization/pre-formed antibodies to HLA antigens	Historical PRA; cPRA derived from identified unacceptable HLA antigens and donor-population frequencies	Higher PRA/cPRA suggests broader sensitization and a lower likelihood of finding an immunologically compatible donor	Highly sensitized candidates may have reduced access to compatible donors and longer waiting times.
Cell-based crossmatch	Presence of recipient antibodies that bind to donor lymphocytes	CDC crossmatch Flow crossmatch	A positive crossmatch indicates donor-reactive antibody-binding/cytotoxicity. CDC crossmatch detects complement-fixing donor-reactive antibodies; flow crossmatch is more sensitive for detecting lower-level donor-reactive antibodies	A positive crossmatch indicates increased risk of AMR and graft loss. A positive T-cell CDC-XM attributable to HLA-DSA generally represents a major immunological barrier to transplantation; interpretation of FCXM depends on antibody specificity, strength, and clinical context. Depending on antibody specificity, crossmatch strength, donor availability and clinical context, alternative donor selection or desensitization may be considered.
Virtual crossmatch	Predicted DSA reactivity based on recipient antibody specificities and donor HLA type	Recipient HLA antibody profile, typically by SAB assay, combined with donor HLA typing	Recipient HLA antibody specificities are compared with donor HLA antigens to determine whether preformed DSA is predicted to be present	Enables rapid assessment of donor–recipient immunological compatibility without physically mixing recipient serum and donor cells; may facilitate organ allocation and, in appropriately selected recipients, transplantation without a prospective physical crossmatch
Antibody characterization	Presence, specificity, and strength of recipient anti-HLA antibodies, including identification of DSAs	Solid-phase HLA antibody assays, particularly Luminex SAB assays	Identifies individual HLA antibody specificities; comparison with donor HLA typing determines whether antibodies are donor specific. MFI provides a semi-quantitative measure of bead fluorescence and should not be interpreted as a direct antibody titer or concentration	Preformed DSA is associated with increased risk of AMR and graft loss. Antibody characterization informs donor selection, virtual crossmatching, immunological risk stratification, and consideration of desensitization or tailored immunosuppression

AMR, antibody-mediated rejection; CDC, complement-dependent cytotoxicity crossmatch; cPRA, calculated panel reactive antibody; DSA, donor-specific antibody; FCXM, flow cytometry crossmatch; HLA, human leukocyte antigen; MFI, mean fluorescence intensity; PRA, panel reactive antibody; SAB, single-antigen bead.

Eplet mismatch represents one of several approaches to quantifying molecular HLA disparity. An alternative is direct assessment of amino-acid mismatch. The HLA Epitope Mismatch Algorithm (HLA-EMMA) compares donor and recipient HLA amino-acid sequences and identifies polymorphic, solvent-accessible amino-acid mismatches potentially accessible to B-cell receptors (20). Unlike HLAMatchmaker, which defines structurally based configurations of polymorphic residues as eplets, HLA-EMMA quantifies molecular disparity directly at the level of accessible amino-acid residues. These related approaches capture overlapping but non-identical aspects of donor–recipient HLA compatibility and should not be considered interchangeable. Their existence also highlights the importance of specifying the molecular mismatch methodology employed when comparing mismatch burdens or proposed clinical thresholds across studies.

### 2.2 Expanding evidence for the prognostic value of molecular mismatch

As molecular histocompatibility assessment has evolved, an expanding body of evidence has demonstrated the prognostic value of molecular mismatch for alloimmune risk and long-term kidney transplant outcomes. In contrast to conventional antigen-level matching, which treats all HLA mismatches equally, molecular approaches characterize donor-recipient HLA disparity with greater resolution, including differences in amino acid sequence and structurally defined eplets.

Early studies largely focused on the quantitative burden of molecular mismatch, demonstrating a relationship between increasing mismatch load and alloimmune outcomes. However, molecular mismatches are unlikely to be uniformly immunogenic, as only a proportion result in detectable antibody responses, suggesting that both the number and characteristics of individual mismatches may contribute to alloimmune risk (18). This has prompted growing interest in moving beyond mismatch quantity to the qualitative immunogenicity of individual HLA mismatches, incorporating their structural, physicochemical, and functional characteristics. Nevertheless, robust assignment of immunogenicity to individual eplets remains challenging, and the relative contributions of mismatch quantity and quality remain active areas of interest and investigation.

Evidence supporting the prognostic relevance of HLA-DR and -DQ molecular mismatch has been particularly consistent. In a retrospective cohort of 664 kidney transplant recipients followed for a median of 7.5 years, Wiebe et al. demonstrated that HLA-DR/DQ single-molecule epitope mismatch was associated with the development of dnDSA, AMR, and Banff ≥1A TCMR (21). Importantly, molecular mismatch provided greater discrimination of alloimmune risk than conventional antigen-level HLA-DR and -DQ mismatch, supporting the additional prognostic information afforded by more granular characterization of donor-recipient HLA disparity.

Subsequent studies have reinforced the particular importance of HLA-DQ molecular mismatch. In a cohort of 926 kidney transplant recipients, Senev et al. demonstrated a linear relationship between the number of HLA-DQ antibody-verified eplet mismatches and the risk of dnDSA but did not identify a threshold below which risk was absent. Eplet mismatches at HLA-DQ are associated with a substantially increased risk of dnDSA formation, graft rejection, and graft failure after kidney transplantation (22). Consistent with these findings, Lim et al. demonstrated that HLA-DQ antigen mismatches associated with acute rejection are also independent of HLA-ABDR mismatches and initial immunosuppressive therapy (23). Collectively, these observations suggest that HLA-DQ molecular disparity represents an important determinant of alloimmune risk following kidney transplant.

The prognostic relevance of molecular mismatch also extends beyond dnDSA and acute rejection to chronic allograft injury and graft survival. In a case-control study, increasing HLA-DR/DQ eplet mismatch burden was associated with transplant glomerulopathy, with a 25% increase in the odds of transplant glomerulopathy for every 10 additional HLA-DR/DQ eplet mismatches (24). More recently, Sapir-Pichhadze et al. demonstrated an independent association between eplet mismatches and death-censored graft failure, providing further evidence that molecular mismatch may serve as a prognostic biomarker for long-term graft outcomes (25).

## 3 Redefining alloimmune risk stratification

As evidence supporting the prognostic importance of molecular mismatch has accumulated, an important question is how to translate it into clinically meaningful risk stratification. In particular, should alloimmune risk be categorized using pre-defined eplet mismatch thresholds, or considered as a continuum across the entire spectrum of molecular mismatch burden? Two influential studies illustrate these complementary approaches. Wiebe et al. identified clinically informative mismatch thresholds that stratified recipients into discrete risk groups, whereas Senev et al. demonstrated a continuous relationship between HLA-DQ eplet mismatch burden and dnDSA development, without identifying a threshold below which risk was absent (21, 22) (Table 2).

**Table 2:** Comparing the approaches of risk stratification.

Comparison domain	Wiebe et al. (2019) (21)	Senev et al. (2020) (22)
Risk relationship identified	HLA-DR ≥7 or HLA-DQ ≥9 eplet mismatches identified as thresholds associated with increased dnDSA development; molecular mismatch provided greater discrimination than conventional antigen-level mismatch	Increasing HLA-DQ eplet mismatch burden demonstrated a continuous relationship with dnDSA development, with no threshold below which risk was absent
Study-derived risk stratification	Single-molecule HLA-DR/DQ eplet mismatch Low risk: HLA-DR <7 and HLA-DQ <9 Intermediate risk: Not meeting low-risk definition and HLA-DQ ≤14 High risk: HLA-DQ ≥15	HLA-DQ antibody-verified eplet mismatch Risk considered along a continuum of HLA-DQ mismatch burden, rather than divided into discrete categories
Key implication	Provides clinically interpretable thresholds that can facilitate risk stratification and potentially guide management and clinical trial design	Avoids assuming a biologically ’safe’ mismatch threshold and captures the progressive increase in alloimmune risk with increasing mismatch burden
Limitations	Categorization may oversimplify a continuous biological relationship; proposed thresholds may not be universally generalizable across all cohorts, loci, outcomes, and mismatch algorithms	Continuous estimates may be less straightforward to translate into discrete clinical decisions or stratification criteria

Importantly, reported molecular mismatch thresholds are method-specific and should not be regarded as interchangeable biological cut-offs. Thresholds may differ by HLA locus, molecular mismatch definition, typing resolution, study population, immunosuppressive regimen, clinical outcome, and analytical approach. Consequently, thresholds derived using single-molecule molecular mismatch, antibody-verified eplet mismatch, or amino-acid mismatch should not be directly extrapolated between methodologies or populations. Cohort-derived thresholds are therefore best regarded as risk-stratification tools that require external validation, rather than universally applicable cut-offs for classifying alloimmune risk.

Threshold-based approaches facilitate classification into low-, intermediate-, and high-risk groups and may provide a pragmatic framework for clinical decision-making and trial design. However, categorization inevitably simplifies the underlying biological relationship, in which alloimmune risk appears to increase progressively with the molecular mismatch burden. These concepts are not mutually exclusive—molecular mismatch may represent a continuous biologic risk factor while clinically derived thresholds provide useful decision points for translating this risk into practice.

More recent work has sought to refine risk assessment beyond the total number of mismatched eplets by considering the immunogenicity of individual molecular mismatches. Schawalder et al. demonstrated substantial heterogeneity in the propensity of individual HLA-DQ eplet mismatches to be associated with antibody formation, suggesting that mismatch quantity alone may incompletely capture alloimmune risk (26). These findings have stimulated efforts to incorporate qualitative characteristics of individual mismatches alongside overall mismatch burden, with the aim of developing more biologically informative and clinically applicable measures of donor-recipient compatibility. Supplementary Table S1 summarizes key studies evaluating the association between molecular mismatch and alloimmune outcomes, including study design, cohort size, follow-up, molecular mismatch methodology, effect estimates, and important limitations.

## 4 Implications for personalized immunosuppression

Current immunosuppressive strategies are informed by clinical and immunological risk factors, yet substantial inter-centre variation in regimen selection persists, often determined by the transplant centre, reflecting the absence of a standardized approach to individualizing immunosuppression (27). Molecular mismatch offers a potential means of refining this assessment by quantifying donor-recipient alloimmune disparity beyond conventional clinical and HLA antigen-level risk factors Figure 3; Table 3 summarizes potential clinical applications of molecular mismatch, distinguishing evidence for prognostic risk stratification from evidence supporting molecular mismatch-guided changes in treatment, surveillance, or donor selection.

**Figure 3:**
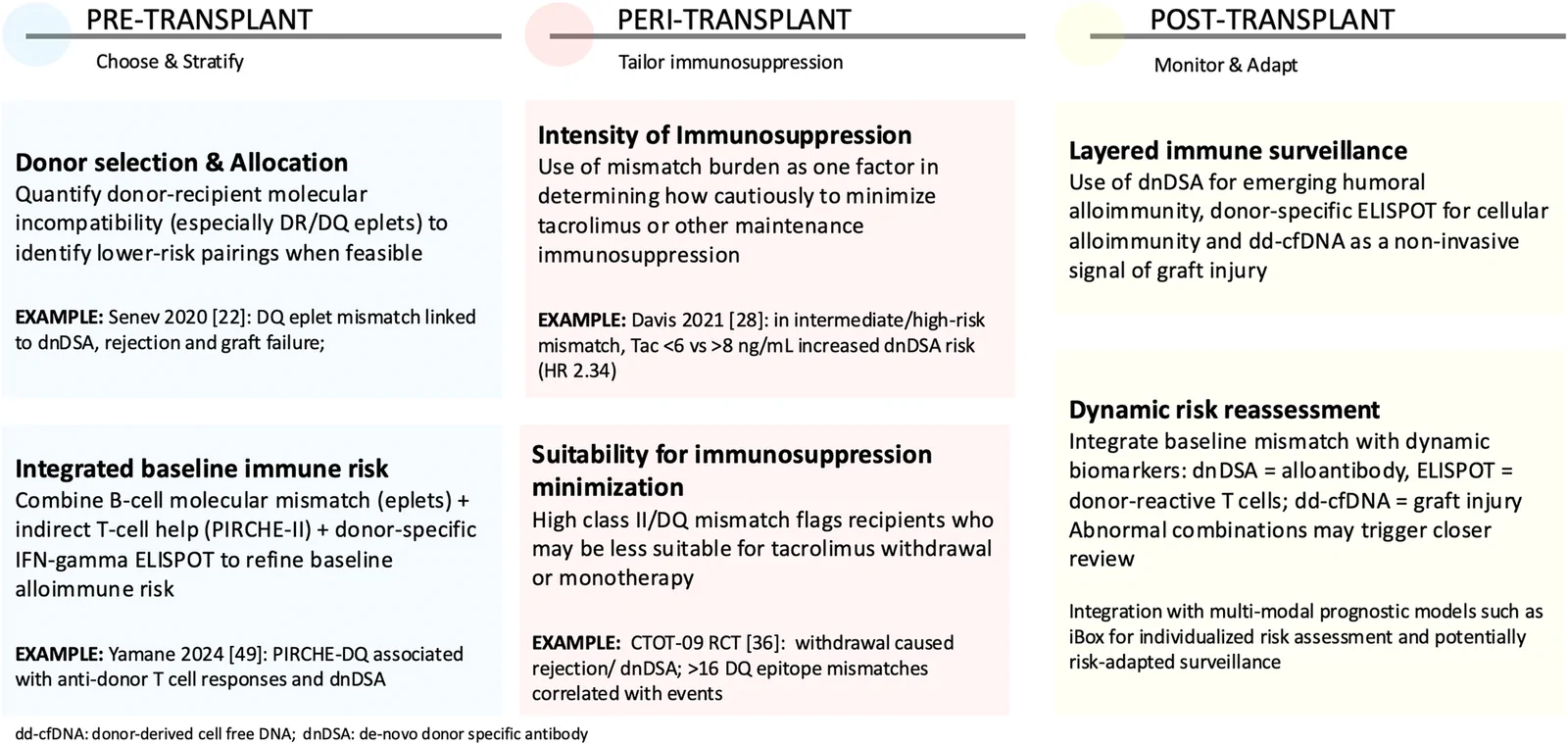
Role of molecular mismatch in kidney transplantation. Conceptual framework illustrating potential applications of molecular mismatch from pre-transplant donor selection and baseline alloimmune risk stratification, through peri-transplant individualization of immunosuppression, to post-transplant immune surveillance and dynamic risk assessment. Pre-transplant molecular HLA compatibility, particularly HLA-DR/DQ eplet mismatch, may be integrated with complementary measures such as PIRCHE-II and functional donor-specific cellular assays to refine baseline alloimmune risk. Molecular mismatch may subsequently identify recipients in whom immunosuppression minimization warrants greater caution, while post-transplant integration with dynamic biomarkers such as *de novo* donor-specific antibodies (dnDSA), donor-specific ELISPOT, donor-derived cell-free DNA (dd-cfDNA), and multivariable prognostic models may support longitudinal reassessment of risk. Selected studies providing supporting evidence for individual components of this framework are shown. Importantly, most evidence remains prognostic or observational, and prospective evidence demonstrating improved outcomes from molecular mismatch-guided treatment, surveillance, or allocation remains limited.

**Table 3:** Potential applications and clinical implementation for molecular mismatch.

Potential application	Evidence supporting risk stratification	Evidence of mismatch-guided intervention	Current clinical status	Key references
Prediction of dnDSA and alloimmune risk	Consistent association between increasing molecular mismatch burden and dnDSA; particularly established for HLA-DR/DQ molecular mismatch	Not applicable to prognostic use itself	Prognostic association established across multiple observational cohorts; methodology and thresholds vary between studies	Wiebe 2019 (21) Senev 2020 (22)
Prediction of rejection and graft outcomes	Molecular mismatch has been associated with TCMR, AMR and graft outcomes, although evidence is strongest for dnDSA and humoral alloimmunity	No prospective evidence that management based on these predictions improves graft survival	Prognostic evidence available; clinical utility of mismatch-guided intervention not established	Wiebe 2019 (21) Senev 2020 (22)
Individualization of maintenance immunosuppression	Observational studies demonstrate that molecular mismatch modifies alloimmune risk associated with reduced tacrolimus exposure; risk also differs across maintenance regimens	No prospective trial has assigned maintenance immunosuppression intensity according to molecular mismatch	Biological rationale and observational evidence; mismatch-guided dosing not established	Wiebe 2017 (31) Davis 2021 (28) Lee 2022 (33) Johnson 2024 (39)
Immunosuppression minimization/withdrawal	Post-hoc analyses of minimization/withdrawal studies suggest molecular mismatch may further discriminate alloimmune risk	Molecular mismatch has not been adequately validated as a prospective treatment-allocation biomarker	Hypothesis-generating evidence; mismatch-guided minimization not established	Hricik 2015 (36) Reinders 2021 (37) Bestard 2021 (53) Bezstarosti 2023 (38)
Risk-adapted dnDSA surveillance	Molecular mismatch can identify groups with substantially different probabilities of developing dnDSA	No prospective trial has demonstrated that altering DSA surveillance frequency according to molecular mismatch improves clinical outcomes	Risk-stratification strategy described; outcome benefit of mismatch-guided surveillance not established	Wiebe 2019 (21) Wiebe 2023 (47)
Selection of induction therapy	Limited observational evidence relating molecular mismatch to outcomes according to induction strategy	No prospective molecular mismatch-guided induction trial	Limited evidence; clinical utility not established	Tran 2025 (40)
Donor selection and organ allocation	Molecular compatibility provides more granular assessment of donor–recipient HLA disparity than conventional antigen matching and is associated with alloimmune outcomes	No prospective evidence that molecular mismatch-based allocation improves net recipient or population outcomes; effects on waiting time and equity require evaluation	Potential application supported by prognostic and modelling evidence; implementation strategy not established	Wiebe 2019 (21) Senev 2020 (22) Niemann 2025 (51)
Multimodal immunological risk assessment	Combining complementary molecular compatibility measures may improve risk discrimination; integration with functional or longitudinal biomarkers is under investigation	No validated prospective strategy has demonstrated improved outcomes from multimodal biomarker-guided management	Emerging evidence; prospective clinical utility not established	Bestard 2021 (53) Niemann 2025 (51) Tian 2026 (52) Kammer 2026 (60)

AMR, antibody-mediated rejection; CNI, calcineurin inhibitor; dnDSA, *de novo* donor-specific antibodies; TCMR, T-cell mediated rejection.

Although clinically validated thresholds and prospective evidence for molecular mismatch-guided treatment remain limited, an emerging body of evidence suggests that the consequences of immunosuppressive exposure may vary according to molecular mismatch burden. Recipients with greater molecular mismatch burden appear particularly susceptible to alloimmunity in the setting of inadequate immunosuppression, whereas those with lower mismatch burden may be less vulnerable to modest reductions in immunosuppressive exposure (28). This interaction raises the possibility of using molecular mismatch to identify recipients who require sustained immunosuppression, and conversely, those in whom minimization might be investigated more safely (29). Importantly, molecular mismatch should therefore be considered a potential tool for risk-adapted immunosuppression rather than an established basis for treatment selection.

### 4.1 Precision molecular mismatch risk-based immunosuppression

Tacrolimus-based regimens remain the cornerstone of maintenance immunosuppression in kidney transplantation. However, prolonged calcineurin inhibitor (CNI) exposure is associated with metabolic and cardiovascular adverse effects, infection, malignancy, and nephrotoxicity, creating a longstanding tension between preventing alloimmunity and minimizing treatment-related toxicity. Identifying recipients in whom immunosuppression can be safely reduced therefore remains an important goal of precision transplant medicine.

Molecular mismatch may provide information beyond conventional clinical risk assessment when considering immunosuppression intensity. Wong et al., in a review of the clinical applications of molecular mismatch, proposed that conventional definitions of low immunological risk may inadequately capture donor-recipient alloimmune disparity and that molecular mismatch could potentially refine selection of recipients for immunosuppression minimization (30).

An important interaction between molecular mismatch and tacrolimus exposure was demonstrated by Wiebe et al. in 596 kidney transplant recipients (31). Both HLA-DR/DQ eplet mismatch and tacrolimus exposure were independently associated with dnDSA development, but the greatest risk occurred among recipients with high molecular mismatch (>11 HLA-DR or -DQ eplet mismatch) and low tacrolimus exposure (<5 ng/mL). In contrast, recipients with low molecular mismatch demonstrated a substantially lower incidence of dnDSA despite lower tacrolimus trough concentrations. These findings suggested that the immunological consequences of reduced CNI exposure may depend on the underlying degree of donor-recipient molecular mismatch.

Subsequent studies have supported this relationship. Lower tacrolimus exposure and reduced time within the therapeutic range have been associated with dnDSA development during the first post-transplant year (32). In 444 donor-recipient pairs, Davis et al. demonstrated an interaction between HLA-DR/DQ molecular mismatch and tacrolimus exposure, with intermediate- and high-risk recipients experiencing greater dnDSA risk at lower tacrolimus concentrations (mean tacrolimus level < 6 ng/mL versus > 8 ng/mL) at one year (28). Similarly, Lee et al. reported a marked increase in dnDSA risk among recipients with both high HLA class II eplet mismatch and low tacrolimus exposure, illustrating the combined effects of molecular mismatch and inadequate immunosuppression (33).

A similar interaction has been observed with medication adherence. In a cohort of 195 kidney transplant recipients, Wiebe et al. demonstrated that the adverse consequences of non-adherence were concentrated among recipients with greater HLA-DQ molecular mismatch (≥17), with substantially higher rates of graft loss (33% vs. 10%) among non-adherent recipients with high molecular mismatch burden (34). These observations support a broader conceptual model in which molecular mismatch represents the underlying alloimmune substrate, while immunosuppressive exposure and adherence modify whether that risk translates into clinically apparent alloimmunity.

These associations should, however, be interpreted cautiously. Immunosuppressive exposure is not randomly assigned in observational cohorts and may be influenced by recipient characteristics, adverse effects, graft function, and other clinical factors that themselves influence alloimmune outcomes. Furthermore, mean or trough calcineurin-inhibitor concentrations do not fully capture intrapatient variability, temporary dose reductions, treatment interruptions, or medication adherence. Non-adherence may simultaneously reduce immunosuppressive exposure and increase alloimmune events, potentially confounding observed relationships between molecular mismatch, drug exposure, and outcomes. The available evidence therefore supports an association between underlying molecular alloimmune susceptibility, immunosuppressive exposure, and alloimmune outcomes, but does not establish that prospectively assigning immunosuppressive intensity according to molecular mismatch improves clinical outcomes.

Immunosuppression reduction may nevertheless be feasible in carefully selected recipients considered low immunological risk by conventional criteria. In a randomized non-inferiority trial of 166 stable kidney transplant recipients more than one year after transplantation, Badell et al. demonstrated that every-two-month belatacept administration was non-inferior to monthly administration for renal function at 12 months (35). Molecular mismatch did not guide participant selection in this study. Rather than providing evidence for mismatch-guided treatment, these findings demonstrate the feasibility of reducing immunosuppressive exposure in selected recipients and raise the question of whether molecular risk assessment could further refine the definition of “low immunological risk.”

The limitations of conventional definitions of “low immunological risk” were illustrated by the CTOT-09 randomized controlled trial, which investigated tacrolimus withdrawal in carefully selected kidney transplant recipients (36). Eligibility for withdrawal required conventional features of low immunological risk, including PRA <30%, absence of DSA, negative flow cytometry crossmatch, stable graft function, absence of rejection, and a stable surveillance biopsy at 6 months. Nevertheless, tacrolimus withdrawal resulted in an unexpectedly high incidence of alloimmune events, with 8 of 14 recipients (57%) developing acute rejection and/or dnDSA, prompting premature termination of the trial. Molecular mismatch was not used for prospective selection; however, subsequent analysis within the study demonstrated an association between HLA-DQ molecular mismatch and alloimmune events following tacrolimus withdrawal. These findings illustrate the limitations of defining “low immunological risk” using conventional clinical and immunologic criteria alone and raise the possibility that molecular compatibility may provide additional information when identifying candidates for immunosuppression minimization.

Similar observations emerged from a sub-study of the TRITON trial examining 29 kidney transplant patients undergoing mesenchymal stromal cell therapy with tacrolimus minimization or withdrawal (37). Recipients who developed dnDSA after tacrolimus withdrawal had a significantly higher HLA-DQ eplet mismatch burden; at a threshold of ≥11 HLA-DQ eplet mismatches, 52% developed dnDSA, compared with none below this threshold (38). Although limited by the small sample size, these findings further support investigating molecular mismatch as a biomarker for selecting candidates for CNI minimization.

Importantly, the association between molecular mismatch and alloimmune risk does not appear restricted to predominantly Caucasian, tacrolimus-treated cohorts. In a predominantly Asian cohort of 234 kidney transplant recipients followed for a median of 5.4 years, Wong et al. demonstrated that HLA-DR/DQ single-molecule mismatch remained associated with alloimmune risk despite substantial differences in maintenance immunosuppression (11). Cyclosporine was used in 71% of recipients, yet only 1% of recipients classified as low molecular mismatch risk (HLA-DR eplet mismatch <7 and HLA-DQ <9) developed dnDSA. These findings support the generalizability of molecular mismatch as a prognostic marker across populations and immunosuppressive regimens, although they do not establish the safety of prospective minimization of immunosuppression.

The relationship between molecular mismatch and alloimmune outcomes may also vary according to immunosuppressive regimen. In a retrospective cohort study, Johnson et al. demonstrated that single-molecule HLA-DR/DQ eplet mismatch stratified the risk of immune events in recipients receiving either conventional tacrolimus-based immunosuppression or Belatacept with time-limited tacrolimus. Importantly, Belatacept with time-limited tacrolimus was associated with lower risks of DSA, AMR, and rejection after stratification by eplet mismatch risk, suggesting that the alloimmune risk associated with molecular mismatch may be modified by the intensity and composition of maintenance immunosuppression (39).

Molecular mismatch may also have potential implications for the selection of induction immunosuppression. Tran et al. proposed that recipients with greater molecular mismatch might benefit from more potent lymphocyte-depleting induction, such as anti-thymocyte globulin, whereas recipients with lower molecular mismatch may be suitable for non-depleting induction with basiliximab (40). However, evidence supporting molecular mismatch-guided induction remains limited, and prospective studies are required before molecular mismatch can be used to select induction therapy in clinical practice.

Collectively, these studies demonstrate that molecular mismatch should not be considered in isolation from immunosuppressive exposure. Recipients with greater molecular incompatibility appear particularly vulnerable to alloimmunity when immunosuppression is inadequate or minimized, whereas recipients with lower molecular mismatch burden consistently demonstrate lower alloimmune risk across a range of immunosuppressive exposures. These observations provide a rationale for prospective evaluation of molecular mismatch-guided immunosuppression, both to identify recipients who may require sustained or more intensive maintenance therapies, and those in whom minimization strategies could potentially be undertaken more safely. This is particularly relevant given the failure of previous immunosuppression-minimization trials using conventional clinical and immunological criteria to reliably identify recipients in whom treatment reduction can be safely undertaken.

### 4.2 Molecular mismatch risk-adapted monitoring and early detection

The development of dnDSA is an important marker of post-transplant alloimmunity and is strongly associated with adverse graft outcomes. A systematic review and meta-analysis of 10,535 kidney transplant recipients across 14 countries found that dnDSA was strongly associated with an increased risk of AMR (a relative risk (RR) of 9.66, 95% confidence interval (CI) 6.79-13.73), acute cellular rejection (RR 2.92, 95% CI 2.16-3.94), chronic antibody-mediated injury and transplant glomerulopathy (RR 6.78, 95% CI 4.31-10.66), and graft loss (RR 4.95, 95% CI 3.81-6.43) (41). Class II dnDSA, particularly those directed against HLA-DQ, account for a substantial proportion of dnDSA and have been consistently associated with chronic rejection and graft failure.

Conventional post-transplant surveillance has largely relied on graft function, with additional DSA testing performed in the setting of graft dysfunction or suspected rejection. However, alloimmune injury may develop before deterioration in serum creatinine becomes apparent. Both dnDSA and histological AMR can therefore be detected in recipients with apparently stable graft function, providing a potential window for identifying alloimmunity before clinically overt graft dysfunction develops (42).

The 2023 European Society for Organ Transplantation consensus report evaluated the role of post-transplant DSA monitoring in recipients with stable graft function. Across the cited studies, among recipients with subclinical DSA detected despite stable graft function, biopsy frequently reveals underlying microscopic injury. Of note, dnDSA was strongly associated with AMR and graft loss. The consensus recognized that DSA may signal underlying histological graft injury and recommended that detection of dnDSA prompt assessment of its pathogenicity and prognosis, with allograft biopsy as the best means of determining the presence and severity of associated injury. Although the optimal surveillance schedule remains uncertain, routine antibody monitoring at three to six months post-transplant and annually thereafter was suggested as a pragmatic approach. Importantly, the consensus emphasized that optimization of maintenance therapy and adherence should follow dnDSA detection, while additional treatment should generally be guided by biopsy findings rather than DSA alone (42–46).

Molecular mismatch may provide an opportunity to move beyond universal surveillance toward risk-adapted monitoring. In a prospective cohort of 949 kidney transplant recipients, Wiebe et al. combined recipient age with HLA-DR/DQ single-molecule mismatch to derive recipient age-molecular mismatch (RAMM) risk categories (47). Approximately 52% of recipients were classified as low risk, among whom subclinical dnDSA-free survival at 5 years was 98% and at 10 years was 97%. In contrast, dnDSA risk increased progressively across intermediate- and high-risk categories, with more than a sixfold difference between the high- and low-risk groups. The authors proposed that RAMM-based surveillance could reduce antibody testing by more than 50% while retaining more intensive surveillance among recipients at greatest risk of dnDSA development.

Molecular mismatch could potentially also be integrated with measures of immunosuppressive exposure. Given the interaction between HLA molecular mismatch, tacrolimus exposure, and dnDSA development, recipients with greater molecular mismatch may warrant closer assessment of tacrolimus exposure and medication adherence, especially when trough concentrations are low or highly variable (33). Together these findings provide a rationale for a dynamic surveillance model in which baseline molecular mismatch defines underlying alloimmune susceptibility, while post-transplant biomarkers—including dnDSA, immunosuppressive exposure, adherence, and graft function—capture the evolving expression of that risk.

### 4.3 Beyond single mismatch scores: towards multimodal alloimmune risk assessment

Molecular mismatch quantifies donor-recipient HLA disparity, but does not, in isolation, capture the multiple biological processes required for an alloimmune response to develop. Although eplet mismatch has been most extensively validated against humoral outcomes such as dnDSA development, structural disparity alone does not determine whether a mismatch will ultimately elicit an antibody response. Generation of a sustained antibody response also requires antigen processing, T-cell recognition, and cognate T-cell help, while the subsequent development of graft injury depends on additional recipient, immunological, and treatment-related factors. Different molecular compatibility approaches may themselves interrogate complementary components of this process: eplet and amino-acid mismatch approaches characterize structural HLA disparity relevant to antibody recognition, Predicted Indirectly Recognizable HLA Epitopes—class II (PIRCHE-II)-based approaches estimate donor-derived HLA peptides that may be presented by recipient HLA class II molecules through the indirect pathway, and non-HLA genomic mismatch captures an additional dimension of donor-recipient genetic disparity beyond the HLA region. Integrating these complementary measures with functional and longitudinal biomarkers may therefore provide a more comprehensive assessment of alloimmune susceptibility than any single metric considered in isolation.

One complementary approach is the PIRCHE-II algorithm, a computational model that estimates the number of donor-derived HLA peptides that can potentially be presented by recipient HLA class II molecules to CD4+ T cells through the indirect pathway of allorecognition. In a large cohort of 2,787 kidney transplant recipients, increasing PIRCHE-II scores were associated with greater risk of dnDSA development and poorer graft survival (48). Subsequent studies have supported the relevance of this pathway. Yamane et al., in a cohort of 105 living-donor kidney transplant recipients, demonstrated higher HLA-DQ PIRCHE-II scores among recipients who developed dnDSA, supporting a relationship between predicted indirect T-cell recognition and humoral alloimmunity (49). Studies combining PIRCHE-II with eplet mismatch have further suggested that these approaches may provide complementary information beyond conventional antigen-level HLA matching (50).

Recent studies have begun to evaluate whether combining complementary molecular compatibility measures improves risk assessment. In a large registry analysis of 400,935 kidney transplants, Niemann et al. compared multiple HLA-derived compatibility metrics, including antibody-verified eplets, amino-acid-based measures, Snow, and PIRCHE-II (51). Multivariable molecular compatibility models generally performed better than individual molecular metrics, and adding molecular compatibility information to established clinical variables provided incremental predictive value, particularly for longer-term outcomes. These findings support a shift from identifying a single optimal mismatch score towards integrating complementary measures of donor–recipient molecular compatibility.

Molecular compatibility algorithms themselves continue to evolve. Tian et al. recently evaluated the updated PIRCHE-T2 model, incorporating the Frost peptide-binding predictor, for dnDSA risk stratification in two independent kidney transplant cohorts (52). The updated model showed associations with dnDSA risk, although performance varied by HLA locus and cohort. These findings emphasize that computational molecular compatibility scores are evolving models rather than fixed biological measurements and reinforce the importance of specifying algorithm version and externally validating proposed risk thresholds before clinical implementation.

An alternative approach combines molecular mismatch with functional measures of donor-specific cellular immunity. The CELLIMIN trial investigated immunosuppression minimization in clinically stable kidney transplant recipients selected using conventional immunological criteria and donor-specific cellular alloreactivity by IFN-*γ* ELISPOT (53). Molecular mismatch was not used to prospectively determine treatment allocation but was evaluated subsequently as an additional risk stratification parameter. Selected recipients underwent withdrawal of mycophenolate mofetil and transitioned to tacrolimus monotherapy. *Post-hoc* analysis demonstrated that HLA class II eplet mismatch provided additional discrimination of alloimmune risk. However, despite prospective selection using clinical and cellular immune criteria, biopsy-proven acute rejection occurred more frequently following minimization than with standard-of-care immunosuppression (25% vs. 11.3% at 1 year). The study was terminated early because of slow recruitment and was underpowered for its primary endpoint. The findings therefore provide hypothesis-generating evidence that molecular and functional measures of alloimmune susceptibility may provide complementary information, rather than prospective evidence supporting molecular mismatch-guided immunosuppression minimization.

These different biomarkers also provide information at different stages of the alloimmune process and should not be considered interchangeable. Molecular HLA compatibility, PIRCHE-based measures and non-HLA genomic mismatch predominantly characterize baseline donor–recipient alloimmune susceptibility and remain largely fixed for a given transplant pair. Immunosuppressive exposure and adherence subsequently modify the probability that this susceptibility becomes clinically expressed. In contrast, dnDSA, donor-derived cell-free DNA (dd-cfDNA), graft dysfunction and histological injury provide dynamic evidence of an evolving alloimmune response or graft injury. Future precision-transplant strategies may therefore be most informative when baseline molecular susceptibility, modifiable treatment exposure and longitudinal biomarkers of immune activation and graft injury are integrated rather than considered in isolation.

Molecular mismatch may also be complemented by dynamic biomarkers of allograft injury. In a prospective observational study of 42 recipients, Gonzalez-Lopez et al. measured dd-cfDNA approximately one month post-transplant and observed higher levels among recipients experiencing acute rejection, particularly AMR (54). Higher HLA-DQ and class II eplet mismatch was also associated with increased dd-cfDNA levels. Although limited by the small cohort and observational design, these findings illustrate the potential complementarity between molecular mismatch and dd-cfDNA: molecular mismatch provides a baseline estimate of alloimmune susceptibility, whereas dd-cfDNA provides a dynamic measure of ongoing allograft injury. Their integration could therefore potentially distinguish recipients predisposed to alloimmunity from those in whom graft injury is actively occurring.

Collectively, these approaches illustrate a potential evolution from single-biomarker risk prediction toward multidimensional alloimmune risk assessment. Molecular mismatch can characterize the baseline antigenic disparity between donor and recipient; PIRCHE-II can estimate the potential for indirect T-cell recognition; functional assays, such as ELISPOT, can detect pre-existing donor-reactive cellular immunity; and dynamic biomarkers, such as dd-cfDNA, can provide evidence of ongoing graft injury. Integration of these complementary measures could ultimately enable risk assessment to evolve from a static pre-transplant estimate to a dynamic model updated throughout the post-transplant course. However, whether multimodal biomarker strategies provide sufficient incremental predictive value and the clinical utility to justify their complexity and cost requires prospective validation.

More precise characterization of donor–recipient compatibility also raises the possibility of incorporating molecular mismatch into donor selection and organ allocation. In principle, preferentially allocating more molecularly compatible organs could reduce alloimmune risk and improve long-term graft outcomes, particularly in recipients with long anticipated graft-survival requirements. Molecular compatibility may also discriminate more effectively than conventional antigen-level matching between donor–recipient combinations that would otherwise appear similarly mismatched.

However, optimizing molecular compatibility must be balanced against the consequences of restricting donor availability. Requiring increasingly stringent molecular matching could prolong waiting time, particularly for recipients with uncommon HLA genotypes or from populations underrepresented in the donor pool, and any incremental reduction in alloimmune risk must be weighed against the competing morbidity and mortality associated with remaining on dialysis. The objective may therefore not be to identify the donor with the lowest possible molecular mismatch for every recipient, but to determine when the expected long-term benefit of improved molecular compatibility justifies declining an otherwise acceptable organ. Prospective allocation modeling and evaluation of effects on equity, waiting time, and graft outcomes will be required before molecular mismatch can be routinely incorporated into deceased-donor allocation.

## 5 Current limitations

Despite the growing evidence supporting HLA-DR/DQ molecular mismatch as a prognostic marker of alloimmune risk, several biological, methodological, and clinical limitations currently constrain its widespread adoption and translation into routine practice.

First, molecular mismatch quantifies potential antigenic disparity but does not directly measure immunogenicity or the resulting immune response. Individual molecular mismatches are not biologically equivalent, and their propensity to elicit alloimmunity may depend on structural and physicochemical characteristics, including amino acid polarity and electrostatic properties (55, 56). Importantly, an eplet represents a computationally defined configuration of polymorphic amino acids on the surface of the HLA molecule and should not necessarily be considered synonymous with an antibody-defined epitope. Consequently, total eplet mismatch burden provides an incomplete representation of the immunogenicity of a donor-recipient combination. Recipients with similar or even identical eplet mismatch scores may experience markedly different outcomes because the development of alloimmunity is also influenced by antigen processing and presentation, pre-existing immune memory, immunosuppression exposure and adherence, infection and inflammatory events, and other donor and recipient factors.

Second, molecular mismatch captures only one component of the biological processes contributing to graft injury. Much of its clinical validation has focused on humoral alloimmune outcomes, particularly dnDSA development and AMR, and its ability to predict other causes of graft dysfunction is less well established. Molecular mismatch would not be expected to directly predict non-alloimmune processes such as CNI toxicity, infection-related graft dysfunction, and other recurrent kidney disease. Jabbour et al., for example, examined sequential protocol biopsies from stable recipients at 3 and 12 months post-transplant and found that progression of chronic histologic lesions during the first year was common but largely unrelated to measures of HLA histocompatibility (57). These findings emphasize that molecular mismatch reflects alloimmune susceptibility rather than a comprehensive predictor of all forms of graft injury.

HLA disparity also does not encompass the entirety of alloimmune recognition. Growing evidence implicates antibodies against non-HLA targets, such as endothelial antigens, in allograft injury, even in recipients without detectable HLA-DSA (58). Genetic disparity outside the HLA region may similarly contribute to graft outcomes. In a prospective cohort study of 477 deceased-donor kidney transplant pairs, greater non-HLA mismatch was independently associated with graft loss after adjustment for HLA eplet mismatch and relevant clinical covariates, with the strongest associations involving mismatches in transmembrane and secreted proteins (HR 1.68 per interquartile-range increase, 95% CI 1.17-2.41) (59). More recent evidence supports the complementary contribution of HLA and non-HLA genomic disparity. Kammer et al. evaluated 1,187 donor–recipient pairs from a prospective kidney transplant cohort and found that genome-wide non-HLA mismatch involving non-synonymous variants in transmembrane or secreted kidney-expressed proteins was associated with graft loss (HR 1.33 per interquartile-range increase, 95% CI 1.02–1.74) (60). Notably, non-HLA genomic mismatch was essentially uncorrelated with HLA eplet mismatch (Spearman r = 0.02), suggesting that the two measures capture largely independent dimensions of donor–recipient disparity. A composite HLA and non-HLA mismatch score was associated with graft loss (HR 1.76, 95% CI 1.20–2.57). These findings support a broader model of histocompatibility in which HLA molecular mismatch is an important but incomplete component of total donor–recipient alloimmune disparity. Emerging computational approaches additionally seek to identify candidate minor histocompatibility antigens from donor–recipient genomic data, although the incremental clinical utility of these approaches remains to be established (61).

Third, substantial methodological heterogeneity remains in how molecular mismatch is quantified and interpreted. Multiple approaches—including eplet mismatch, antibody-verified eplet mismatch, amino-acid mismatch, and T-cell epitope prediction—interrogate different aspects of donor-recipient HLA disparity, and no universally accepted algorithm or threshold for clinical risk stratification currently exists. Molecular mismatch assessment may also differ by the resolution of the underlying HLA typing, whether high-resolution typing is directly available or inferred through imputation, and the algorithm or database version used. These methodological differences should therefore be considered when comparing molecular mismatch estimates across studies. This is particularly relevant as computational models continue to evolve and increasingly incorporate multiple measures of molecular compatibility. Proposed thresholds also vary according to HLA locus, population, outcome, and analytical approach (33). Moreover, evidence that alloimmune risk increases continuously with molecular mismatch burden challenges the concept of a universal boundary separating low- from high-risk transplantation. Thresholds remain useful for clinical implementation but require external and prospective validation and should not be interpreted as absolute biological boundaries.

Fourth, the generalizability of the existing evidence requires further evaluation. Many influential molecular mismatch studies have been conducted in predominantly Caucasian populations receiving tacrolimus-based maintenance immunosuppression. Although associations have subsequently been demonstrated in other populations and immunosuppressive contexts, including a predominantly Southeast Asian cohort in which cyclosporine was the principal CNI (11), broader validation across ancestrally diverse populations, different donor types, and contemporary immunosuppressive regimens remains important.

More importantly, the existing evidence base is predominantly observational, and prognostic validity has not yet translated into established clinical utility. Studies vary in cohort size, follow-up, immunosuppressive regimens, DSA surveillance, biopsy practices, and outcome definitions, while analyses of less frequent clinical outcomes may be limited by small event counts and imprecise effect estimates. Furthermore, few prospective interventional studies have evaluated whether assigning surveillance or immunosuppressive strategies according to molecular mismatch improves outcomes. Importantly, improved prognostic discrimination does not itself establish clinical utility. For molecular mismatch assessment to guide management, prospective studies must demonstrate not only that it identifies recipients at differential risk, but also that modifying immunosuppression, surveillance, or donor selection based on this information improves clinically meaningful outcomes without disproportionate infectious, malignant, metabolic, or access-related consequences. This distinction is particularly important because current evidence supporting molecular mismatch as a prognostic biomarker is considerably stronger than evidence supporting molecular mismatch-guided intervention.

Finally, molecular mismatch is an inherently static pre-transplant characteristic, whereas alloimmune risk evolves throughout the transplant course. Changes in immunosuppressive exposure and adherence, infection, inflammation, dnDSA development, and other immunological events may substantially modify the expression of baseline molecular mismatch risk. Molecular mismatch may therefore ultimately be most informative when incorporated into dynamic risk models alongside post-transplant biomarkers rather than used as a stand-alone measure. Its potential applications to other aspects of transplant practice, including induction therapy and donor allocation, remain comparatively underexplored.

## 6 Future direction and conclusions

HLA-DR/DQ molecular mismatch represents an important advance in transplant immunology, providing a more granular assessment of donor-recipient immunological disparity than conventional antigen-level HLA matching. A growing body of evidence supports its prognostic value for dnDSA development, rejection, and long-term graft outcomes, while emerging data suggest that molecular mismatch may also help identify recipients particularly vulnerable to inadequate immunosuppression. These findings provide a compelling rationale for incorporating molecular mismatch into precision transplant medicine, although evidence that mismatch-guided treatment improves clinical outcomes remains limited.

Future research should therefore prioritize prospective interventional trials to evaluate whether molecular mismatch-guided immunosuppression can improve the balance between the prevention of alloimmunity and treatment-related toxicity. Standardization of molecular mismatch algorithms, clinically meaningful and actionable thresholds, and reporting methods will also be essential for broader implementation. Beyond maintenance immunosuppression, comparatively little is known about whether molecular mismatch can guide induction therapy or the selection of immunosuppressive agent classes. Molecular mismatch could also potentially inform donor allocation, although any gains in immunological compatibility would need to be carefully balanced against waiting time, equity, and access to transplantation.

A further opportunity lies in integrating molecular mismatch with dynamic, multidimensional risk-prediction models. Tools such as the iBox prognostic system combine clinical, functional, immunological, and histological parameters to estimate the evolving risk of graft failure (62). Molecular mismatch and dynamic prognostic models provide conceptually distinct yet potentially complementary information—molecular mismatch characterizes the baseline immunogenetic disparity between donor and recipient, whereas longitudinal models, such as iBox, estimate the evolving trajectory of graft risk after transplantation. Future approaches incorporating molecular mismatch alongside dynamic biomarkers, clinical variables, and computational prediction models may therefore provide a more comprehensive framework for longitudinal risk assessment and personalized surveillance and treatment.

Although much of the evidence for molecular mismatch has originated in kidney transplantation, its application is increasingly being investigated in other solid-organ transplants, including heart and lung transplantation (63, 64). This broader applicability supports the biological relevance of measuring HLA molecular mismatch across transplantation, while also emphasizing the need for organ-specific validation of molecular mismatch algorithms, thresholds, and clinical applications.

Molecular mismatch represents an important advance beyond conventional antigen-level HLA matching and provides reproducible prognostic information regarding alloimmune risk, particularly the development of dnDSA and antibody-mediated injury. However, molecular mismatch is unlikely to function optimally as an isolated biomarker. Emerging evidence suggests that integration of complementary HLA compatibility metrics, non-HLA genomic disparity, immunosuppressive exposure, and longitudinal biomarkers of alloimmune activation and graft injury may provide a more complete representation of individual transplant risk. The next step for the field is therefore not simply greater precision in measuring donor–recipient mismatch, but prospective demonstration that this information can be translated into safer and more effective individualized surveillance and immunosuppression. Until such evidence is available, molecular mismatch should be regarded primarily as a prognostic tool and framework for understanding alloimmune susceptibility, rather than a stand-alone determinant of clinical management.
