# Supplementary material for: HLA molecular mismatch – A step towards precision medicine in transplantation for individualized immune risk assessment and immunosuppression
Source: Front Med (Lausanne). 2026 Oct 23;13:1990225. doi: 10.3389/fmed.2026.1990225 (PMC13740249; doi:10.3389/fmed.2026.1990225)
Supplement: Supplementary file 1 [file Table1.docx]

**Supplementary Table 1: Key studies evaluating the association between molecular mismatch and alloimmune outcomes**

| **Study** | **Design/ Cohort** | **Number** | **Follow Up** | **Mismatch measure** | **Outcome** | **Main finding/ Effect Estimate** | **Key limitations** |
| --- | --- | --- | --- | --- | --- | --- | --- |
| Wiebe et al., 2019 [21] | Retrospective single-centre cohort | 664 kidney transplant recipients | Median 91 months | Traditional whole antigen method; HLAMatchmaker DRDQDP to determine eplet mismatch sum; HLAMatchmaker to determine single molecule eplet mismatch | Development of dnDSA | AUC 0.84 for HLA-DR/DQ single-molecule eplet mismatch, compared with 0.54–0.58 for conventional whole-antigen mismatch.  Recipients could be stratified into low, intermediate and high molecular mismatch risk categories | Small sample size in a single centre; mostly tacrolimus-treated cohort; requires external validation |
| Senev et al., 2020 [22] | Single-centre observational cohort | 926 kidney transplant recipients | Median 7.45 years | Next generation sequencing of 11 HLA loci, use of HLAMatchmaker to count eplet mismatch | Development of dnDSA,  TCMR/ABMR, eGFR decline, graft failure | Each HLA-DQ antibody-verified eplet mismatch increased risk of DQ dnDSA by 30% (HR 1.30, 95% CI 1.18-1.44), rejection by 6% (OR 1.06, 95% CI 1.03-1.09), AMR by 12% (OR 1.12, 95% CI 1.04-1.20) and graft failure by 5% (HR 1.05, 95% CI 1.01-1.09) | Single centre; 98% Caucasian population; retrospective study; unable to generalise to other populations |
| Sapir-Pichhadze et al., 2015 [24] | Nested case-control study | 52 kidney transplant recipients with biopsy proven transplant glomerulopathy; 104 matched controls | Controls matched on similar time from transplant; duration not retrieved | HLAMatchmaker | Biopsy-proven transplant glomerulopathy | OR 1.25 (95% CI 1.04-1.50) for every 10 additional eplet mismatches; OR 2.84 (95% CI 1.03-7.84) for 27-43 eplet mismatch; OR 4.62 (95% CI 1.51-14.14) for >43 eplet mismatches compared with <27 | Small sample size; DR and DQ eplet mismatch burden largely considered quantitatively; not able to establish causality; proposed mismatch thresholds require external validation |
| Sapir-Pichhadze et al., 2020 [25] | Retrospective observational cohort study | 118,382 unsensitized US first transplants | Not reported | Antibody-verified eplet mismatches (imputed HLA-A/B/C/DRB1/DQB1) | Death-censored graft failure | Every 10 additional antibody-verified eplet mismatch increased death-censored graft failure for class I (HR 1.231, 95% CI 1.195-1.268), DRB1 (HR 1.268, 95% CI 1.231-1.305) and DQB1 (HR 1.411, 95% CI 1.331-1.495) | Retrospective observational study; uncertain biological significance of antibody-verified eplets |
| Wiebe et al., 2017 [31] | Retrospective cohort with serial tacrolimus levels | 596 recipients; 50,011 tacrolimus troughs |  | HLAMatchmaker | Class II dnDSA | Eplet mismatch and low tacrolimus exposure independently predicted dnDSA; highly mismatched recipients are more vulnerable when tacrolimus trough <5 ng/mL | Observational study cohort; potential confounding by medication adherence; limited generalisability |
| Davis et al., 2021 [28] | Single-centre observational validation | 444 kidney transplant donor-recipient pairs | 1-year post-transplant | Wiebe single-molecule DR/DQ thresholds into low-, intermediate- or high-risk | Development of dnDSA within 1 year | AUC 0.84 (DR), 0.82 (DQ)  Intermediate-risk recipients had HR 15.39 (95% CI 2.01-118.09) and high-risk recipients had HR 23.81 (95% CI 3.17-178.66) for DR/DQ dnDSA  Among intermediate/ high-risk recipients, mean tacrolimus <6 vs >8 ng/mL increased dnDSA risk (HR 2.34, 95% CI 1.05-5.22) | Small sample size; few DSA events; short follow-up of 1 year; tacrolimus exposure not randomised |
| Hricik et al., 2015 [36] | Prospective, randomized, multi-centre trial | 21 kidney transplant recipients | Planned 24 months; trial terminated early | HLA-DQ epitope mismatch burden | Change in IFTA between implantation and planned 24-month protocol biopsy | Tacrolimus withdrawal was associated with clinically evident acute rejection in 4/14 (29%) and dnDSA in 5/14 (36%); overall, 8/14 (57%) experienced acute rejection and/or dnDSA. HLA-DQ epitope mismatch >16 was associated with development of dnDSA and/or acute rejection. | Small sample size; terminated early; mismatch analysis post hoc; highly selected low-risk living-donor recipients |
| Bezstarosti et al., 2023 [38] | Post-hoc of randomized phase II TRITON trial | 56 kidney transplant recipients | 2 years | HLA-DQ eplet mismatch load (NGS second field typing); thresholds ≥11 total, ≥8 antibody-verified | Development of dnDSA | Each additional HLA-DQ eplet mismatch increased hazard of dnDSA (HR 1.07, 95% CI 1.02-1.13); ≥11 DQ eplets predicted dnDSA in all MSC patients but specificity 44% | Small sample size; limited generalisability; short follow-up for outcomes |
| Wong et al., 2024 [11] | Observational cohort study | 234 kidney transplant recipients | Median 5.4 years | HLAMatchmaker  Wiebe single-molecule DR/DQ categories, plus cohort-specific thresholds | Development of HLA-DR/DQ DSA | 12% developed HLA-DR/DQ dnDSA; eplet mismatch risk categories predicted dnDSA-free survival (p = 0.001) | Small sample size; observational design; Singapore cohort; main endpoint dnDSA rather than rejection or graft loss |
| Johnson et al., 2024 [39] | Retrospective single-centre cohort study | 588 kidney transplant recipients | 3 years | Wiebe single-molecule DR/DQ categories from imputed HLA typing | Development of DSA, AMR, overall rejection | After stratification by eplet mismatch risk, Bela/TacTL was associated with lower DSA (HR 0.42, 95% CI 0.20–0.88), AMR (HR 0.19, 0.05–0.64), and rejection (HR 0.45, 0.31–0.66) compared with tacrolimus; no difference in death or graft loss (HR 0.96, 0.58–1.59). | Retrospective single-centre propensity-score-matched study; residual confounding possible; 3-year outcome assessment |
| Bestard et al., 2021 [53] | Multicentre biomarker-guided randomized trial | 167 enrolled; 101 ELISPOT-negative recipients randomized (LI n=48; SOC n=53); 66 ELISPOT-positive recipients received SOC | 6-month primary endpoint | Preformed donor-reactive T-cell immunity; HLA eplet mismatch | 6-month BPAR (primary); development of dnDSA | At 6 months, BPAR occurred in 13% with tacrolimus monotherapy versus 2% with SOC (p=0.15). In post-hoc analysis of ELISPOT-negative recipients undergoing minimization, BPAR occurred in 40% with high versus 14% with low class II eplet mismatch risk (p=0.043); eplet mismatch was also associated with anti-DQ dnDSA (p<0.001) | Terminated early and underpowered; eplet analysis post hoc; full rejection rates not seen |

AMR, Antibody-mediated rejection; AUC, area under the curve; Bela, belatacept; DSA, donor-specific antibody; BPAR, biopsy-proven acute rejection; dnDSA, de-novo donor-specific antibody; IFTA, interstitial fibrosis/tubular atrophy; MSC, mesenchymal stromal cell therapy; SOC, standard of care; TacTL, time-limited tacrolimus; TCMR, T-cell mediated rejection;
